# FOXP3-based immune risk model for recurrence prediction in small-cell lung cancer at stages I–III

**DOI:** 10.1136/jitc-2021-002339

**Published:** 2021-05-18

**Authors:** Minlin Jiang, Chunyan Wu, Liping Zhang, Chenglong Sun, Hao Wang, Yi Xu, Hui Sun, Jun Zhu, Wencheng Zhao, Qiyu Fang, Jia Yu, Peixin Chen, Shengyu Wu, Zixuan Zheng, Yayi He, Caicun Zhou

**Affiliations:** 1 Department of Medical Oncology, Shanghai Pulmonary Hospital, Tongji University Medical School Cancer Institute, Tongji University School of Medicine, No 507 Zhengmin Road, Shanghai 200433, China; 2 Tongji University, No 1239 Siping Road, Shanghai 200433, China; 3 Department of Pathology, Shanghai Pulmonary Hospital, Tongji University Medical School Cancer Institute, Tongji University School of Medicine, No 507 Zhengmin Road, Shanghai 200433, China

**Keywords:** tumor microenvironment, biomarkers, tumor, lung neoplasms, lymphocytes, tumor-infiltrating, programmed cell death 1 receptor

## Abstract

**Background:**

Immunotherapies may prolong the survival of patients with small-cell lung cancer (SCLC) to some extent. The role of forkhead box protein P3 (FOXP3) in tumor microenvironment (TME) remains controversial. We aimed to examine FOXP3-related expression characteristics and prognostic values and to develop a clinically relevant predictive system for SCLC.

**Methods:**

We enrolled 102 patients with histologically confirmed SCLC at stages I–III. Through immunohistochemistry, we determined the expression pattern of FOXP3 and its association with other immune biomarkers. By machine learning and statistical analysis, we constructed effective immune risk score models. Furthermore, we examined FOXP3-related enrichment pathways and TME traits in distinct cohorts.

**Results:**

In SCLC, FOXP3 level was significantly associated with status of programmed death-ligand 1 (PD-L1), programmed cell death protein 1 (PD-1), CD4, CD8, and CD3 (p=0.002, p=0.001, p=0.002, p=0.030, and p<0.001). High FOXP3 expression showed longer relapse-free survival (RFS) than the low-level group (41.200 months, 95% CI 26.937 to 55.463, vs 14.000 months, 95% CI 8.133 to 19.867; p=0.008). For tumor-infiltrating lymphocytes (TILs), subgroup analysis demonstrated FOXP3 and PD-1, PD-L1, lymphocyte activation gene-3, CD3, CD4, or CD8 double positive were significantly correlated with longer RFS. We further performed importance evaluation for immune biomarkers, constructed an immune risk score incorporating the top three important biomarkers, FOXP3, TIL PD-L1, and CD8, and found their independently prognostic role to predict SCLC relapse. Better predictive performance was achieved in this immune risk model compared with single-indicator-based or two-indicator-based prediction systems (area under the curve 0.715 vs 0.312–0.711). Then, relapse prediction system integrating clinical staging and immune risk score was established, which performed well in different cohorts. High FOXP3-related genes were enriched in several immune-related pathways, and the close relationships of interleukin-2, *CD28*, basic excision repair genes *MUTYH*, *POLD1*, *POLD2*, and oxidative phosphorylation related gene cytochrome c oxidase subunit 8A with FOXP3 expression were revealed. Moreover, we found low-immune risk score group had statistically higher activated CD4^+^ memory T cells (p=0.014) and plasma cells (p=0.049) than the high-risk group. The heterogeneity of tumor-infiltrating immune cells might represent a promising feature for risk prediction in SCLC.

**Conclusion:**

FOXP3 interacts closely with immune biomarkers on tumor-infiltrating cells in TME. This study highlighted the crucial prognostic value and promising clinical applications of FOXP3 in SCLC.

## Introduction

Among diverse cancers, lung cancer ranks the first in morbidity and mortality, posing a growing threat to human health.^1^ Small-cell lung cancer (SCLC), which accounts for approximately 10%–15% of cases, is a cancer phenotype with high recurrence rate and growth fraction, resulting in poor outcomes.^2 3^ The programmed cell death protein 1 (PD-1) and programmed death-ligand 1 (PD-L1) inhibitors have been confirmed effective in treating non-small-cell lung cancer (NSCLC). They can prolong the survival of patients with SCLC in combination with the standard first-line chemotherapy to some extent.^4 5^ In IMPOWER-133, clinical survival was longer in patients receiving etoposide/carboplatin/atezolizumab than the control group.^4^ The phase 3 CASPIAN study in SCLC showed longer overall survival (OS) when adding PD-L1 inhibitors to chemotherapy.^5^ However, compared with patients with NSCLC, patients with SCLC did not get great benefit from immune-checkpoint inhibitors. CheckMate 331 indicated that patients after progress did not benefit from immunotherapy.^6^ Although the predictive ability of PD-L1 expression has been verified in NSCLC, few predictive biomarkers are available in SCLC. Thus, it is important to explore other targets and immune biomarkers for SCLC.

Forkhead box protein P3 (FOXP3), belonging to the forkhead/winged-helix family, is of great importance in modulating the differentiation and development of T-regulatory cells (Tregs).^7 8^ The numbers of FOXP3^+^ Tregs as well as exhaustive subsets of CD4^+^ and CD8^+^ T cells have been observed increased in solid tumor tissues of patients with colorectal and breast cancers, which helps to create an immunosuppressive environment and promotes tumor progression.^9 10^ In addition, checkpoints such as lymphocyte activation gene-3 (LAG-3), T-cell immunoglobulin and mucin domain-containing protein 3 (TIM-3), and cytotoxic T lymphocyte-associated antigen-4 (CTLA-4) were coexpressed mainly by tumor-infiltrating FOXP3^+^Helios^+^ Tregs, whereas little was detected coexpressed by Tregs with negative expression of FOXP3.^11^ Tumor-infiltrating lymphocytes (TILs) have important prognostic values in solid tumors, reflecting the ability of immune response and predicting patients’ response to a specific anticancer therapy. The correlation between the presence of FOXP3^+^ Tregs and patients’ survival has been extensively studied in many cancer types,^12–17^ which remains conflicting. Therapeutic approaches of targeting Tregs could enhance CD8^+^ TILs and increase antigen presenting cell (APC) function, which may become as a potential strategy in many cancers.^18 19^ In this study, we determined the expression pattern of FOXP3 on TILs and its association with other checkpoints or markers in SCLC through immunohistochemistry (IHC) staining. We also connected the immune status with patients’ survival of SCLC in patients with different FOXP3 levels and constructed effective immune risk models to predict relapse of SCLC. Furthermore, we examined FOXP3-related enrichment pathways, crucial genes, and immune microenvironment characteristics using bioinformatics analysis.

## Methods

### Patient samples

SCLC samples of 102 subjects were collected from January 2014 to December 2018 in Shanghai Pulmonary Hospital, China. We used the eighth edition of tumor, node, metastasis classification for lung cancer for stage identification and prognosis evaluation.^20^

### IHC for FOXP3

For all formalin-fixed, and paraffin-embedded slides, xylene and alcohol were used for dewaxing, followed by aqua destillata for rinsing. Next, we used the target retrieval solution (Dako, DM828/DM829) and recovered antigens under high pressure and heat. To decrease background staining, 0.3% hydrogen peroxide was used. We applied purified anti-human FOXP3 (1:100, BioLegend 320101) as primary antibody and goat-anti-rabbit/anti-mouse IgG that was conjugated with horseradish peroxidase as secondary antibody for testing.

### Determination of IHC cut-off for FOXP3

Two pathologists (CW and LZ) reviewed and determined clinically pathological samples independently. Disagreements were resolved by consensus in these two reviewers. The cut-off value of FOXP3 level on TILs was defined as ≥1% staining. We performed survival analysis for cut-off determination.^21 22^

### eXtreme Gradient Boosting (XGBoost) and immune risk scoring system

We adapted machine learning algorithm XGBoost for importance evaluation and immune risk model construction.^23^ In addition to discovering non-linear relationships by working with data of the first and second derivatives, this machine learning method also controls the overfitting as well as overcomplexity of one predictive model by employing regularization item. XGBoost algorithm can also score the importance of each attribute as value measure.

In this study, we repeated 1000 times for cohort division and model construction to make full use of the sample information. The whole 102 samples were divided into training set and calibration set (7:3) randomly. By incorporating all immune biomarkers, the XGBoost-based model was constructed and the top three features were selected for further survival prediction. Then, we used the calibration set to validate the model performance and performed receiver operating characteristic (ROC) curve analysis for visualizing its predictive value. To construct immune risk assessment system for patients with SCLC, we combined the importance outcomes that ranked the top three of XGBoost model and the coefficients of multivariate Cox regressions that included these immune biomarkers. The final equation was calculated: immune risk score=(−0.067×FOXP3)−(0.023×PD-L1 on TILs)+(0.002×CD8). Further, we confirmed the prognostic role of immune risk score in SCLC and compared its predictive performance with single-indicator-based and two-indicator-based immune risk score systems.

### Nomogram algorithm and relapse model for outcome prediction

The entire set was randomly divided into the training and validation set (n=71 and n=31, respectively) by r. Based on the independently predictive factors of multivariate Cox regression analyses, we adopted nomogram algorithm for relapse prediction in SCLC. After establishing the nomogram, the calibration plots for relapse-free survival (RFS) of 1, 3, and 5 years graphically demonstrated the correlation between predicted and observed risks of each outcome for appraising the predictive ability of this model. To fully test its performance, the nomogram was subjected to 100 bootstrap resamples in the training cohort for internal verification. We also used C-index and ROC curve analyses of 1, 3, and 5 years to assess its discriminating ability.

To reveal the clinical value of this prognostic nomogram, we further regrouped all subjects and made low-risk, moderate-risk, and high-risk stratifications. Survival analyses were conducted for each group and were compared by log-rank test.

### Clinical value of the FOXP3 expression and risk model

For further assessing the clinical value of FOXP3 in patients with SCLC, the clinical data from cBioportal Database were used (https://www.cbioportal.org). We screened samples based on the inclusion criteria^24^: (1) mRNA sequencing (mRNA-seq) of SCLC tissues, (2) complete data for mRNA expression, and (3) complete prognostic information from patients with SCLC.

### Verification of the expression of FOXP3, PD-L1, and CD8 in SCLC

To figure out the expression of *FOXP3*, *CD274* that encodes PD-L1 protein and *CD8A* that encodes CD8 protein in SCLC tissues, we used the Gene Expression Omnibus (GEO) Database for verification (https://www.ncbi.nlm.nih.gov/geo/). mRNA expression data enrolled must follow the inclusion criteria: (1) complete data for mRNA expression, (2) mRNA-seq of SCLC tissues, and (3) mRNA-seq of normal tissues. The exclusion criteria were as follows: (1) insufficient data for comparing gene expression, (2) sample from animals, and (3) mRNA-seq for SCLC cell lines.

### Gene set enrichment analysis (GSEA)

To investigate relevant biological pathways between distinct FOXP3 expression status in SCLC, GSEA software V.4.1.0 was applied.^25^ Based on FOXP3 level, mRNA expression dataset in the GEO was divided into high and low expression groups. The number of gene set permutations was 1000 times, and phenotype label was set as ‘high expression versus low expression’. GSEA analysis according to correlation coefficient (CC) was also conducted. In our study, the absolute values of a normalized enrichment score (NES) of >1 and a false discovery rate (FDR) q value of <0.25 were considered as meaningful GSEA sets.

### Tumor-infiltrating immune profiles in patients with SCLC

We used the method of CIBERSORT as a tool to analyze the immune landscape of patients with SCLC in the tumor microenvironment (TME). On the bases of linear support vector regression and deconvolution, CIBERSORT is an online database to analyze immune infiltration of tumor tissues through 22 immune-cell phenotypes.^26^ Leukocyte signature matrix (LM22), which contains source data and gene expression matrix, includes 547 genes distinguishing 22 hematopoietic cell types of human: naïve and memory B cells, plasma cells, seven T-cell phenotypes, myeloid subsets, and NK cells. We divided 23 SCLC samples into low-immune risk and high-immune risk groups, which was on the basis of the expression levels of FOXP3, PD-L1, and CD8. After that, we investigated the correlations among distinct immune cells and revealed the heterogeneity of tumor-infiltrating immune cells in theses SCLC samples between two immune risk groups.

### Statistical analysis

The correlation between different clinical factors and FOXP3 expression level was appraised by Chi-square tests. By Spearman rank correlation test, we also investigated the correlations of FOXP3 levels with other immune biomarkers. Then, considering distinct clinical characteristics, we conducted univariate as well as multivariate logistic regression analyses to predict FOXP3 expression in patients with SCLC. In order to evaluate prognosis condition, we also used Kaplan-Meier and Cox regression method for survival analysis. Pearson correlation test was used for continuous variables. p<0.05 was defined as statistical significance. Data analysis and visualization were conducted based on statistical tool SPSS V.26.0, GraphPad Prism V.7, and R Programming Language (R V.3.6.1).

## Results

### Patients’ characteristics

One hundred and two subjects with SCLC were included (online supplemental table 1). The baseline information of clinical characteristics among these patients with SCLC were summarized (online supplemental table 2). The median age was 63.5 years (lower limit:38; upper limit: 81). Most subjects were male (84, 82.4%) and 18 (17.6%) were female. There are slightly more non-smokers (58, 56.9%) than smokers (44, 43.1%). All patients enrolled were in clinical staging I–III, and 98 patients (96.1%) did not develop tumor metastasis.

10.1136/jitc-2021-002339.supp1 Supplementary data

### FOXP3 expression on TILs and its relationship with clinical data and other markers

**Figure 1:**
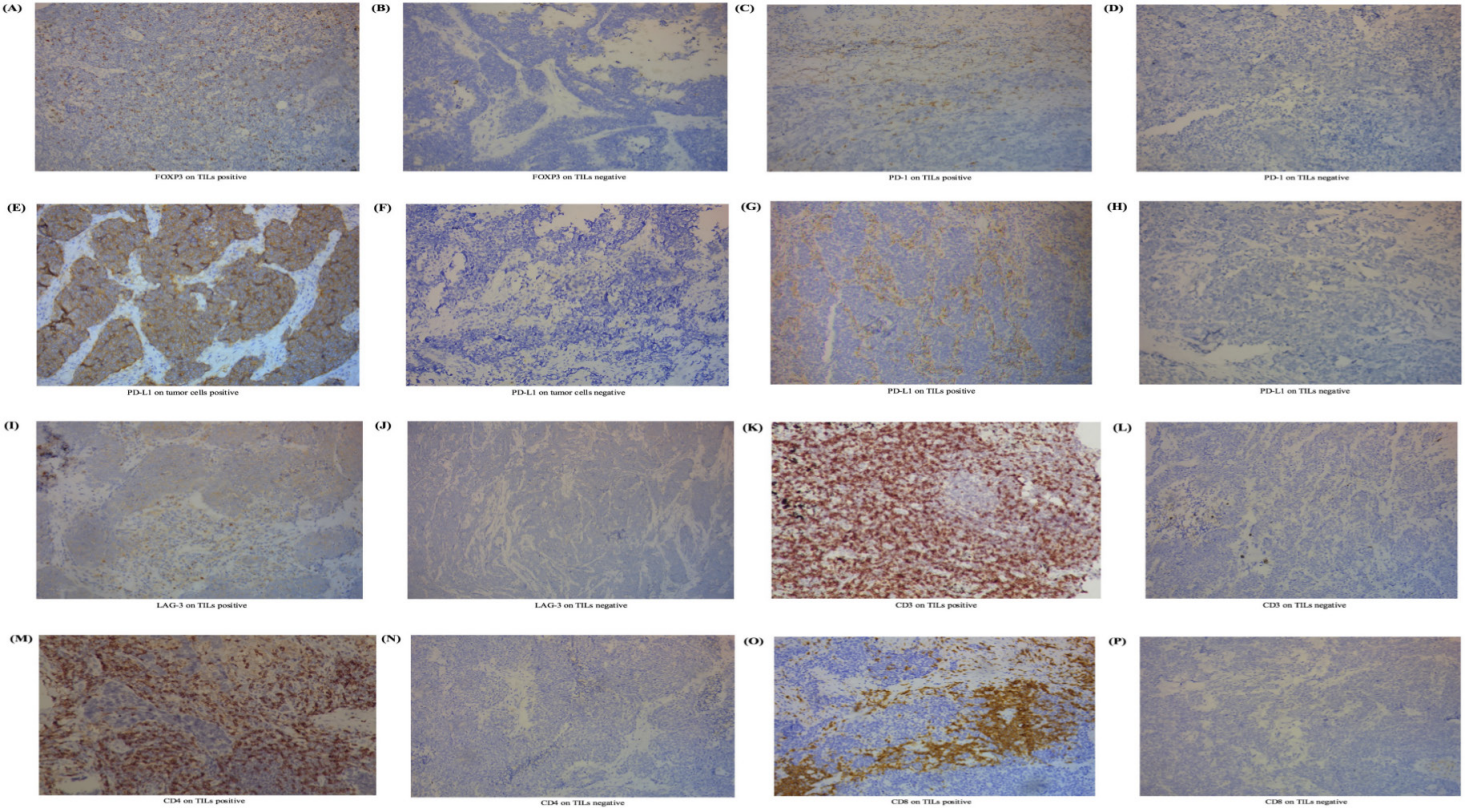
Expression level of FOXP3 on TILs and other immune biomarkers in SCLC. (A) IHC positive for FOXP3 on TILs. (B) IHC negative for FOXP3 on TILs. (C) IHC positive for PD-1 on TILs. (D) IHC negative for PD-1 on TILs. (E) IHC positive for PD-L1 on tumor cells. (F) IHC negative for PD-L1 on tumor cells. (G) IHC positive for PD-L1 on TILs. (H) IHC positive for PD-L1 on TILs. (I) IHC positive for LAG-3 on TILs. (J) IHC negative for LAG-3 on TILs. (K) IHC positive for CD3 on TILs. (L) IHC negative for CD3 on TILs. (M) IHC positive for CD4 on TILs. (N) IHC positive for CD4 on TILs. (O) IHC positive for CD8 on TILs. (P) IHC positive for CD8 on TILs. The process to calculate the cut-off point was completed through survival analysis.^21 22^ Cut-off values for FOXP3, PD-1, PD-L1, LAG-3, CD3, CD4, and CD8 on TILs were defined as 1%, 1%, 5%, 20%, 40%, 30%, and 30%. For tumor cells, PD-L1s of <50% were viewed as negative in this study. FOXP3, forkhead box protein P3; IHC, immunohistochemistry; LAG-3, lymphocyte activation gene-3; PD-1, programmed cell death protein 1; PD-L1, programmed death-ligand 1; SCLC, small-cell lung cancer; TIL, tumor-infiltrating lymphocyte.

We performed IHC and pathological test and revealed the expression of biomarkers on both TILs and SCLC cells (figure 1). In this study, FOXP3 expression was tested positive in 88 samples on TILs (figure 1) (86.3%, ≥1% staining), while 14 (13.7%) did not. The positive expression of PD-1 was 41 (40.2%) on TILs and 0 (0.0%) on tumor cells. Two patients (2.0%) tested positive for the expression of PD-L1 on SCLC cells and 38 (37.3%) tested positive for the expression of PD-L1 on TILs. Expression of FOXP3 on TILs was significantly associated with clinical staging (p=0.013). We observed no correlation between FOXP3 expression on TILs and clinical data including age (p=0.320), gender (p=0.689), smoking status (p=0.077), metastasis (p=0.504) and chemotherapy (p=0.360, online supplemental table 3).

The connection of FOXP3 levels and immune checkpoints or other immune markers was analyzed (online supplemental table 4). Interestingly, we found that FOXP3 expression on TILs was closely associated with checkpoints including PD-1 on TILs (CC=0.327, p=0.001), as well as PD-L1 on TILs (CC=0.307, p=0.002). Furthermore, FOXP3 level was also positively correlated with CD3 (CC=0.366, p<0.001), CD4 (CC=0.307, p=0.002), and CD8 levels (CC=0.215, p=0.030). Regretfully, FOXP3 levels showed no correlation with PD-L1 on SCLC cells (p=0.573). We failed to investigate the correlation between FOXP3 expression and PD-1 levels on tumor cells for the limited samples.

### Logistic regression for FOXP3 expression

We performed both univariate and multivariate logistic analysis for FOXP3 expression. After adjusting relevant parameters, the analysis results were summarized in online supplemental table 5. The ORs for FOXP3 expression on TILs were 4.375 (95% CIs 1.268 to 15.091, p=0.019) for patients at stages I and II compared with those at stage III, and 0.051 (95% CI 0.006 to 0.406, p=0.005) when comparing specimens with positive CD3 on TILs with those with negative CD3.

### Investigation of the correlation between FOXP3 levels and RFS status in SCLC

**Figure 2:**
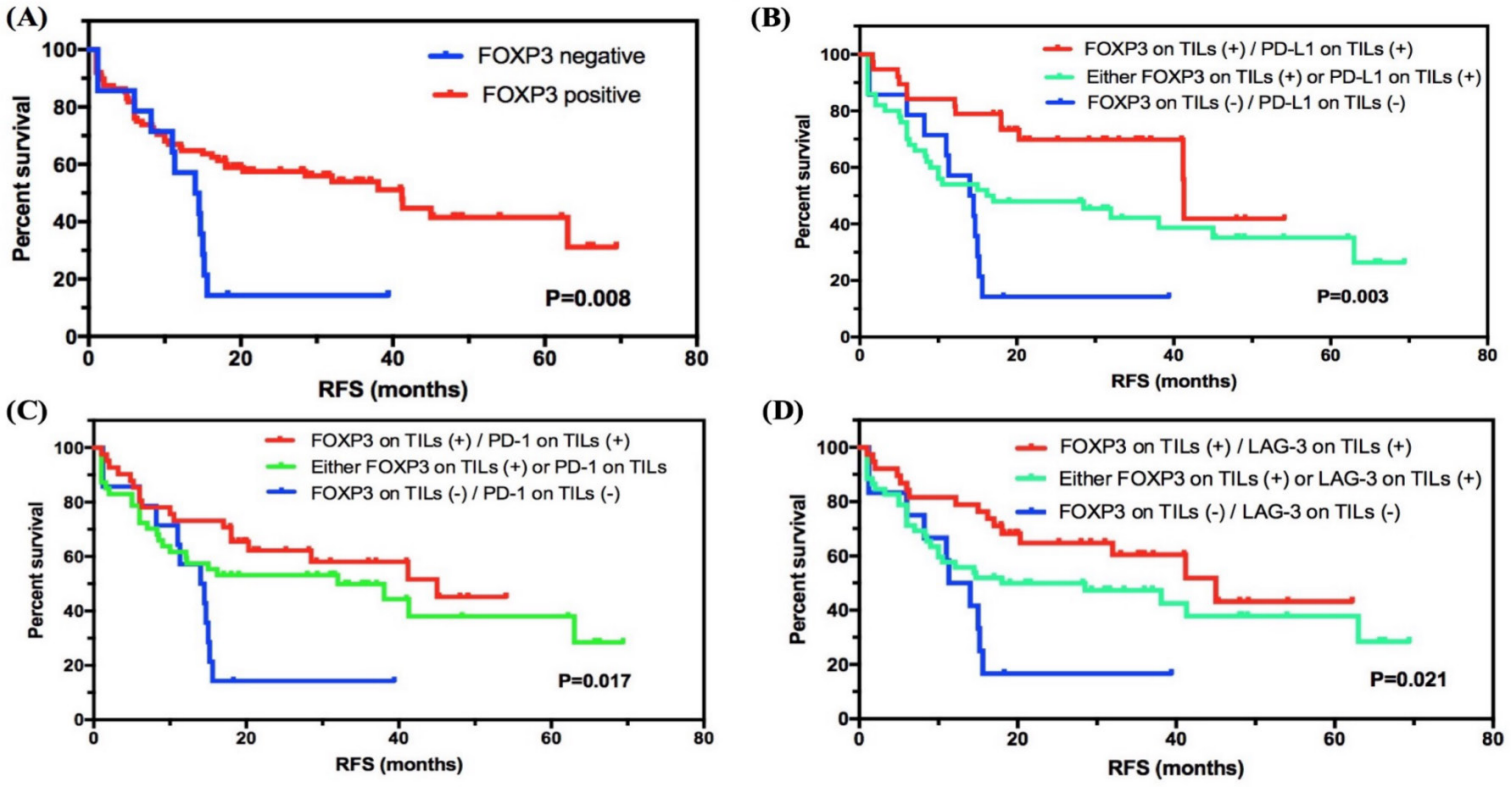
Survival analyses of FOXP3 level on TILs in SCLC. (A) FOXP3 level on TILs was significantly correlated with RFS status in SCLC, and the positive group showed a higher survival compared with the negative group. (B–D) Subgroup survival analyses of FOXP3 expression in combination with checkpoints PD-L1, PD-1, or LAG-3. FOXP3, forkhead box protein P3; LAG-3, lymphocyte activation gene-3; PD-1, programmed cell death protein 1; PD-L1, programmed death-ligand 1; RFS, relapse-free survival; SCLC, small-cell lung cancer; TIL, tumor-infiltrating lymphocyte.

In all subjects involved, the median RFS of 102 patients was 32.000 months (95% CI 12.106 to 51.894), and 56/102 (54.9%) patients had relapses. For 60 patients (60/102, 58.8%) with stage I–II SCLC, 25 (25/102, 41.7%) relapsed (median RFS: 63.000 months, 95% CI 26.271 to 99.729). For 42 patients (42/102, 41.2%) with stage III SCLC, 31 (73.8%) had the end event (median RFS: 14.7000 months, 95% CI 10.360 to 19.040), which showed a significantly lower RFS compared with patients with stage I–II SCLC (p=0.004, online supplemental table 6). We then investigated the correlation between FOXP3 levels and RFS status in SCLC. Interestingly, FOXP3 level on TILs was significantly correlated with RFS status in SCLC, and the positive group showed longer RFS compared with the negative group (41.200 months, 95% CI 26.937 to 55.463, vs 14.000 months, 95% CI 8.133 to 19.867; p=0.008; figure 2A).

We then combined diverse expression patterns based on FOXP3 levels and performed subgroup analysis (figure 2B–D and online supplemental figure 1). Importantly, for immune checkpoints expressed on TILs, FOXP3 and PD-L1 double positive (vs either FOXP3 or PD-L1 positive, or FOXP3 and PD-L1 double negative; 41.300 months, 95% CI 41.076 to 41.524, vs 16.200 months, 95% CI 0.000 to 38.336, vs 14.000 months, 95% CI 8.133 to 19.867; p=0.003), FOXP3 and PD-1 double positive (vs either FOXP3 or PD-1 positive, or FOXP3 and PD-1 double negative; 45.000 months, 95% CI undefined, vs 32.000 months, 95% CI 0.926 to 63.074, vs 14.000 months, 95% CI 8.133 to 19.867; p=0.017), and FOXP3 and LAG-3 double positive (vs either FOXP3 or LAG-3 positive, or FOXP3 and LAG-3 double negative; 45.000 months, 95% CI 27.333 to 62.667, vs 18.000 months, 95% CI 0.000 to 44.807, vs 11.300 months, 95% CI 6.208 to 16.392; p=0.021) showed a statistical correlation with longer RFS. Besides, for other immune biomarkers, FOXP3 and CD3 double positive (vs either FOXP3 or CD3 positive, or FOXP3 and CD3 double negative), FOXP3 and CD8 double positive (vs either FOXP3 or CD8 positive, or FOXP3 and CD8 double negative), and FOXP3 and CD4 double positive (vs either FOXP3 or CD4 positive, or FOXP3 and CD4 double negative) also showed higher survival with statistical significance (p=0.005, p=0.002, and p=0.001). For checkpoints expressed on tumor cells, the subgroup analysis of combing FOXP3 on TILs as well as PD-L1 level on SCLC cells revealed a significant prognostic value as well (p=0.017). However, the sample size of checkpoint expression in tumor cells was too limited. Further investigations are required.

10.1136/jitc-2021-002339.supp2 Supplementary data

### Cox regression analysis for RFS in SCLC

The univariate analysis suggested that clinical stage (p=0.006; HR=0.474, 95% CI 0.279 to 0.804) and FOXP3 levels on TILs (p=0.011; HR=2.352, 95% CI 1.221 to 4.531) were predictive factors for RFS in SCLC. By multivariate COX regression, we found that clinical stage (I–II vs III–IV; p=0.016, HR=0.516, 95% CI 0.301 to 0.886) and FOXP3 on TILs (negative vs positive; p=0.041, HR=2.008, 95% CI 1.027 to 3.927) were considered as independently predictive factors (table 1).

**Table 1:** Univariate and multivariate Cox regression analysis of relapse-free survival

Variables	Univariate			Multivariate			Multivariate		
	HR	95% CI	P value	HR	95% CI	P value	HR	95% CI	P value
Age (<65 y vs ≥65 years)	0.608	0.355 to 1.040	0.069						
Sex (female vs male)	1.676	0.757 to 3.707	0.203						
Smoking status (non-smoker vs smoker)	0.591	0.345 to 1.010	0.054						
T (1–2 vs 3–4)	0.987	0.480 to 2.031	0.972						
N (0 vs 1–3)	0.649	0.372 to 1.134	0.129						
M (0 vs 1)	1.140	0.277 to 4.682	0.856						
Stage (I–II vs III–IV)	0.474	0.279 to 0.804	**0.006**	0.516	0.301 to 0.886	**0.016**	0.478	0.281 to 0.814	**0.007**
FOXP3 on TILs (negative vs positive)	2.352	1.221 to 4.531	**0.011**	2.008	1.027 to 3.927	**0.041**			
Immune risk score (low vs high)	0.505	0.293 to 0.872	**0.014**				0.512	0.296 to 0.884	**0.016**

Data with statistical significance were highlighted in bold.

FOXP3, forkhead box protein P3; M, metastasis; N, node; T, tumour; TIL, tumor-infiltrating lymphocyte.

### Immune risk model construction by XGBoost and its validation

**Figure 3:**
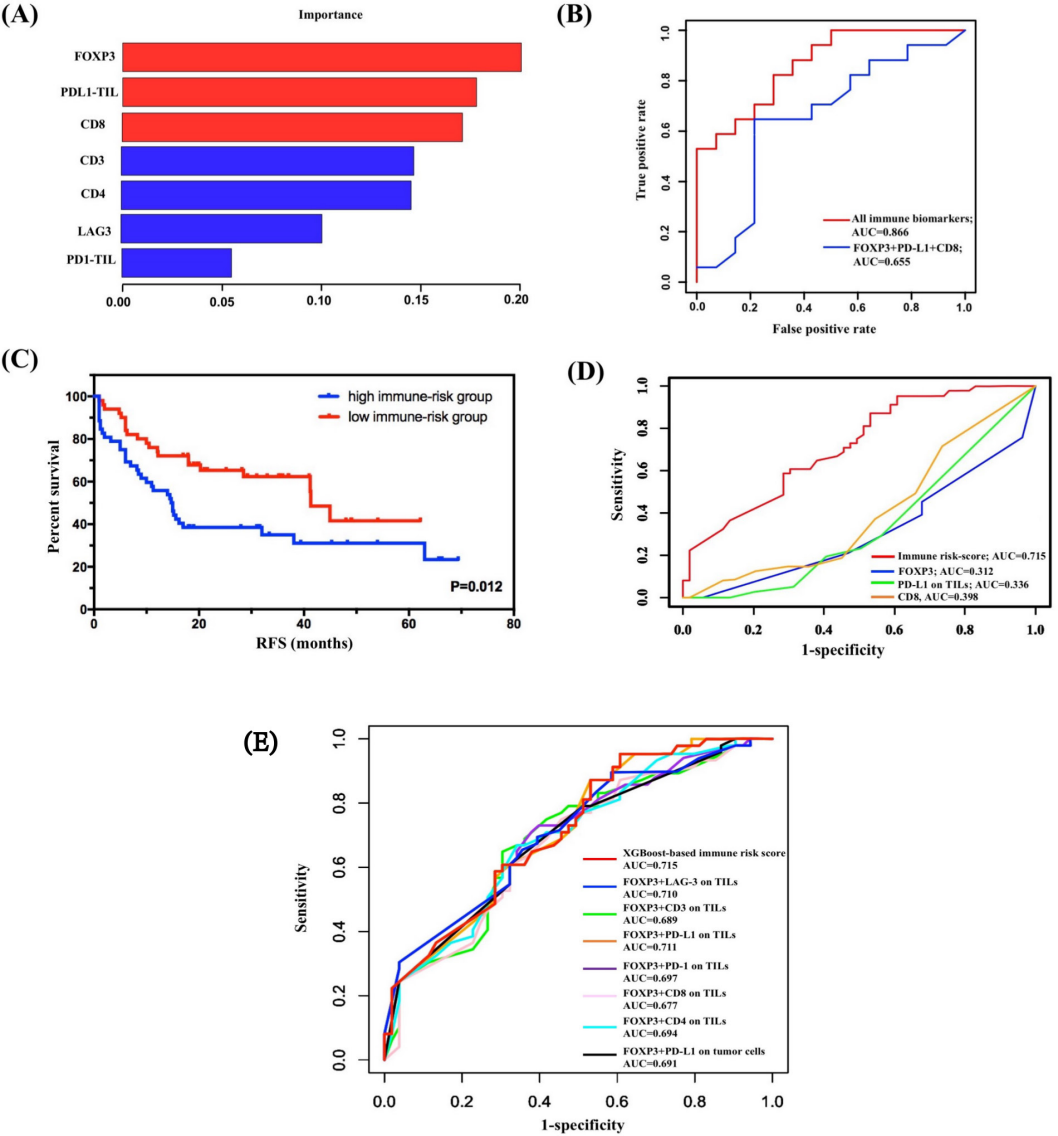
Construction and validation of immune risk score system by XGBoost. (A) Considering all immune indicators, the importance feature map demonstrated the first rank of FOXP3 on TILs, followed by PD-L1 on TILs and CD8. (B) Validation of the model performance by ROC curve analysis in the calibration set. The machine learning model by XGBoost matched the actual one well. (C) Survival analysis on the basis of the immune risk score (RFS high-immune risk group vs low-immune risk group, 14.700 months, 95% CI 10.107 to 19.293, vs 41.300 months, 95% CI 35.773 to 46.827; p=0.012). (D) Comparison of the predictive performance between immune risk score and single indicators using time-dependent ROC curve analysis. (E) Comparison of the predictive performance between immune risk score and two-indicator based immune risk systems using time-dependent ROC curve analysis. AUC, area under the curve; FOXP3, forkhead box protein P3; PD-L1, programmed death-ligand 1; RFS, relapse-free survival; ROC, receiver operating characteristic; TIL, tumor-infiltrating lymphocyte.

The subgroup analysis revealed great significance of FOXP3 levels on TILs in combination with other immune markers to prognosis evaluation in SCLC. Thus, we hypothesized that FOXP3 levels on TILs connected closely with other immune molecules. We used XGBoost algorithm for importance assessment and feature selection to validate this assumption. By repeating 1000 times considering all immune parameters, the importance feature map demonstrated the first rank of FOXP3 on TILs, followed by PD-L1 on TILs and CD8 (figure 3A). To validate the model performance, we performed ROC curve analysis in the calibration set. The machine learning model by XGBoost matched the actual one well, with an area under the curve (AUC) of 0.866 (figure 3B). Further, integrating the first three biomarkers also showed well-predictive ability (AUC=0.655, figure 3B).

Given the importance results of the XGBoost model, FOXP3, PD-L1 on TILs, and CD8 were selected as variables for construction of immune risk model in SCLC. As shown in the survival diagram by Kaplan-Meier analysis, patients with SCLC with high-risk scores (52/102, 51.0%) had poorer prognosis (vs patients with SCLC with low-risk scores; 14.700 months, 95% CI 10.107 to 19.293, vs 41.300 months, 95% CI 35.773 to 46.827; p=0.012; figure 3C). We further conducted univariate Cox regression, and the regression result revealed a statistically predictive role of immune risk score in RFS of SCLC (HR=0.505, 95% CI 0.293 to 0.872; p=0.014). Given that FOXP3 on TILs was incorporated in the construction of immune risk score, a multivariate Cox regression model of RFS was built based on SCLC staging and immune risk score. Both SCLC staging (HR=0.478, 95% CI 0.281 to 0.814; p=0.007) and immune risk score (HR=0.512, 95% CI 0.296 to 0.884; p=0.016) were independently and significantly correlated with RFS in SCLC (table 1).

To further confirm the prognostic role of immune risk score in SCLC, we compared the predictive performance between immune risk score and single indicators at first (figure 3D). The AUC values of time-dependent ROC analyses showed this immune risk score system performed better than single biomarkers including FOXP3, PD-L1 on TILs, and CD8 (figure 3D). Also, considering the significant survival differences of subgroup analysis based on FOXP3 and TIL PD-L1, TIL PD-1, TIL LAG-3, CD3, CD4, CD8, or PD-L1 on tumor cells, we established seven unique immune risk scores using multivariate Cox analysis and compared their performance with XGBoost-based risk score. All combined immune systems showed good performance (figure 3E). Among distinct combined biomarkers, the XGBoost-based immune risk score obtained the best AUC value of 0.715, which performed better than either biomarkers analyzed previously in predicting relapse of SCLC at stages I–III (figure 3E).

### Predicted probability of stage I–III SCLC relapse

**Figure 4:**
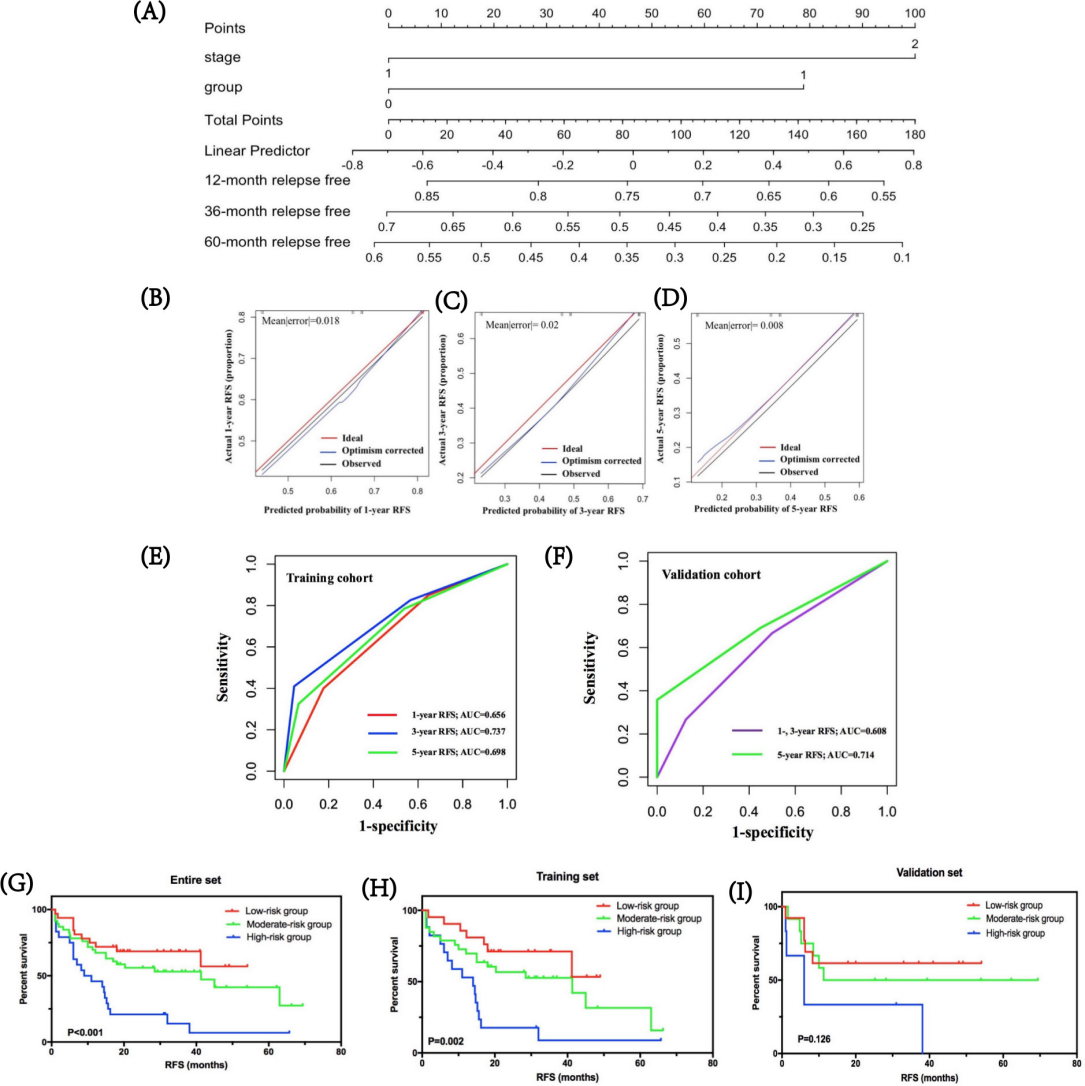
Predicted probability of stage I–III SCLC relapse. (A) by integrating immune risk score and SCLC staging, nomogram algorithm was used to predict the probability of RFS in SCLC of 1, 3, and 5 years. (B–D) Calibration plots for the probability of RFS of 1, 3, and 5 years. (E, F) Time-dependent ROC curves for risk model in the training and validation cohorts. The AUC values for RFS of 1, 3, and 5 years were 0.656, 0.737, and 0.698, respectively, in the training cohort, and 0.608, 0.608, and 0.714, respectively, in the validation cohort. (G–I) The clinical values of the prognostic nomogram and survival analyses of three distinct risk groups in the entire SCLC cohort, training set, and validation set. AUC, area under the curve; RFS, relapse-free survival; ROC, receiver operating characteristic.

By integrating immune risk score and SCLC staging, we used nomogram algorithm to predict the probability of RFS of 1, 3, and 5 years in SCLC using training dataset. The nomogram indicated the immune risk score contributed slightly less to patients’ prognosis compared with clinical stage (figure 4A). Calibration plots and C-index were conducted for the probability of RFS of 1, 3, and 5 years, and the C-index was 0.639 (figure 4B–D). Furthermore, we assessed and validated its effectiveness by time-dependent ROC analysis. The AUC values for RFS of 1, 3, and 5 years were 0.656, 0.737, and 0.698, respectively, in the training cohort, and 0.608, 0.608, 0.714, respectively, in the validation cohort (figure 4E, F), which highlighted that the predictive model performed well in predicting relapse of patients with SCLC in stages I–III.

Based on predictive scores, the cut-off values were determined by regrouping all subjects in the entire SCLC cohort, as well as the training and validation cohorts into three subgroups with distinct prognosis. Survival analyses showed that in the entire SCLC, training, and validation cohorts, the low-risk group had the highest 1-year RFS at 81.1%, 86.4%, and 67.6%, respectively, followed by the moderate-risk group at 67.0%, 75.5%, and 48.6%, respectively. The high-risk group suggested the lowest 1-year RFS for the entire, training, and validation cohorts: 44.4%, 52.4%, and 27.2%, respectively (figure 4G–I). Further, the low-risk groups in all cohorts were correlated with higher RFS compared with moderate-risk and high-risk groups (p<0.001 in the entire SCLC cohort, p=0.002 in the training cohort, and p=0.126 in the validation cohort, respectively).

### Clinical value of FOXP3 expression and risk model in SCLC

We used cBioportal Database^27^ to retrieve SCLC clinical datasets and downloaded the suitable one with 81 samples. After screening gene panels, RNA-seq data of patients with SCLC were available for nine samples (online supplemental table 7). Then, we investigated the coexpression correlation between protein FOXP3 and other immune biomarkers (online supplemental figure 2A). The results showed some similar correlations. Significant significance was analyzed in the correlation of FOXP3 expression with the expression of PD-1, LAG-3, CD3, and CD4 (p=0.001, p=0.014, p=0.002, and p=0.015, respectively) (online supplemental table 8). Among them, PD-1 expression showed the highest relevance (CC=0.894). In addition, the results demonstrated no significant correlation of FOXP3 expression with the expression of PD-L1 (p=0.584) and CD8 (p=0.977). For the relationship between FOXP3 and clinical data, the mRNA expression of *FOXP3* had no statistical association with diagnosis age (p=0.206) as well as gender (p=0.379). Further, we identified the relationship between FOXP3 expression and patients’ prognosis in SCLC (online supplemental figure 2B). The survival analysis showed that the group with high FOXP3 expression had longer survival compared with the group with low FOXP3 expression (17.000 months, 95% CI 2.300 to 31.700, vs 10.000 months, 95% CI 1.180 to 18.820; p=0.076). We also extended the risk model to the prognosis evaluation of these patients with SCLC (online supplemental figure 2C). The survival results indicated that patients with SCLC with high risk showed shorter OS compared with the low-risk and moderate-risk groups (10.000 months vs 17.000 months, p=0.603).

10.1136/jitc-2021-002339.supp3 Supplementary data

### Corroboration of expression of FOXP3, PD-L1 and CD8 in the SCLC cohort

For better understanding of the relative expression of mRNA *FOXP3*, *CD274* (PD-L1), and *CD8A* (CD8) in SCLC, we verified their levels, respectively, in an SCLC cohort. After retrieving gene expression of SCLC, the GSE43346 dataset that included 23 SCLC samples and 42 normal tissues was chosen available for analysis. For FOXP3 expression, significant difference between SCLC tissues and normal lung controls (p=0.0061 in GSE43346 dataset) was calculated (online supplemental figure 3A). For PD-L1 (*CD274*), the GEO cohort also suggested a significant difference of expression between SCLC tissues and control subjects (p=0.034 in GSE43346 dataset) (online supplemental figure 3B). For CD8, there was no significant difference shown in GSE43346 dataset (p=0.8; online supplemental figure 3C). In particular, the expression of FOXP3 in SCLC tissues was lower than that of the normal group; and PD-L1 expression in SCLC tissues was higher than that of the controls.

10.1136/jitc-2021-002339.supp4 Supplementary data

**Figure 5:**
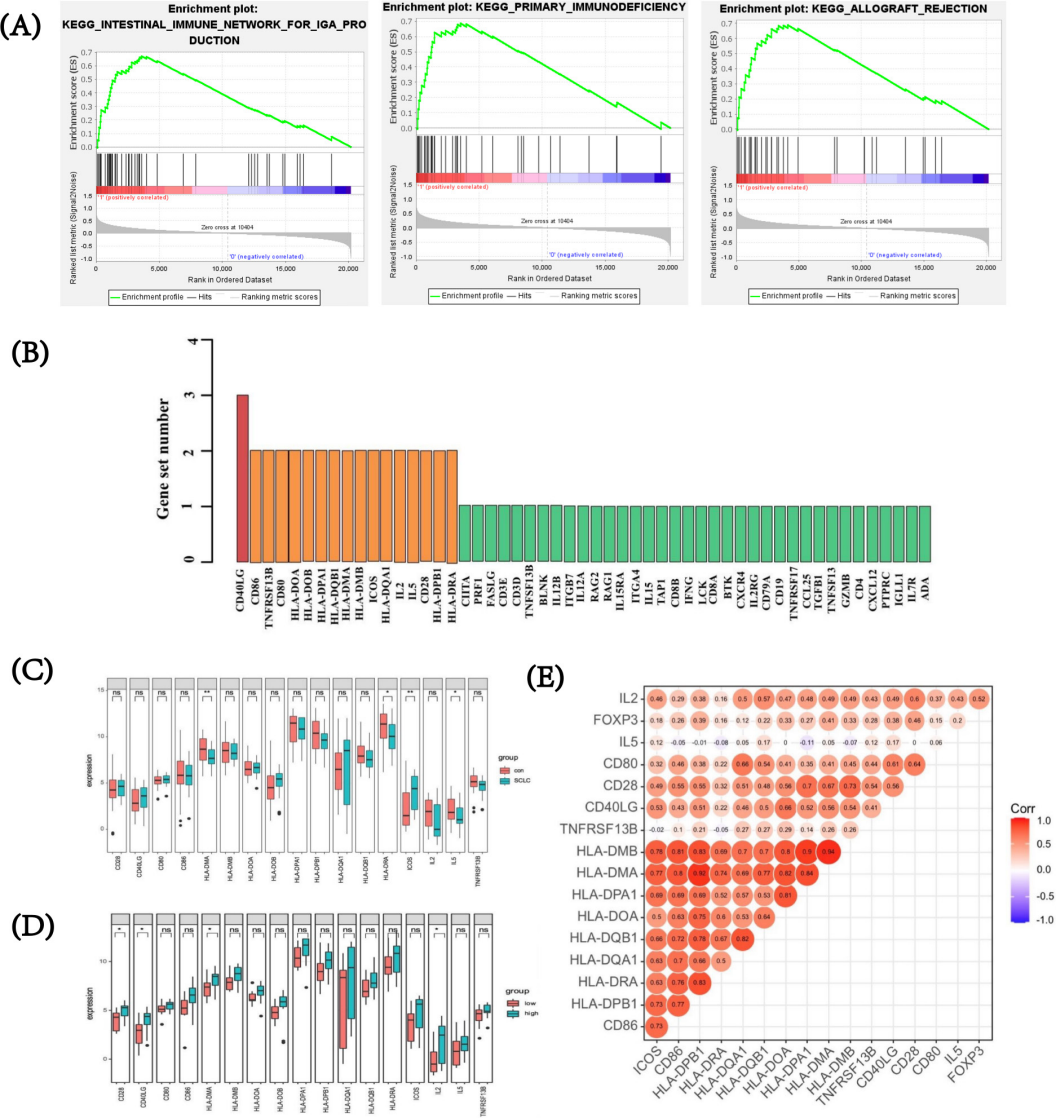
FOXP3 expression, relevant biological pathways, and putative targets by gene set enrichment analysis in SCLC. (A) Top three pathways for high FOXP3 expression group. (B) Overlapping rate and close correlations among the top three high FOXP3 expression correlated pathways. (C) After verifying expression levels of the overlapped genes, four showed a significant difference between SCLC tissues and normal specimens. (D) Expression of overlapped genes between high and low FOXP3 expression groups. (E) Relationship between the expression of *FOXP3* and overlapped genes. FOXP3, forkhead box protein P3; SCLC, small-cell lung cancer.

To investigate relevant biological pathways and potential regulatory genes related to FOXP3 expression in SCLC, we further performed GSEA in the GSE43346 dataset to excavate putative targets related to FOXP3. The whole cohort was divided into high and low FOXP3 expression groups according to the median value of FOXP3 expression. In 174 gene sets that were upregulated or downregulated between the FOXP3 high and low expression groups, 108 gene sets upregulated in the SCLC group with high FOXP3 expression (108/174, 62.1%) were analyzed, while 66 gene sets upregulated in the group with low FOXP3 expression (66/174, 37.9%) were analyzed. High FOXP3 expression-related genes were enriched in several immune-related pathways such as intestinal immune network for IgA production (|NES|=2.300, FDR q value<0.001), primary immunodeficiency pathway (|NES|=2.267, FDR q value<0.001), and cytokine–cytokine receptor interaction (|NES|=2.158, FDR q value<0.001). The top three FOXP3 high expression-related pathways were depicted, with the absolute value of NES >1 and FDR q value<0.25 (figure 5A). Figure 5B demonstrated a high overlapping rate and close correlations among the top three high FOXP3 expression correlated pathways. Specifically, through leading edge analysis, a total of 17 genes were found important in at least two pathways (17/53, 32.1%), and *CD40LG* overlapped in all of these pathways. After verifying expression levels of the aforementioned overlapped genes, four showed significant differences between SCLC tissues and normal specimens (p<0.05) (figure 5C). Further, by Benjamini and Hochberg multiple testing to correct p value, inducible co-stimulator (ICOS), with the absolute value of log2-fold change of >1, was found differentially expressed between SCLC and normal tissues. By t-test, overexpression of *CD28*, *CD40LG*, *HLA-DMA*, and interleukin (IL)-2 was observed in high FOXP3 expression group (all p<0.05, figure 5D). In addition, we conducted a correlation analysis and found that FOXP3 was positively associated with IL-2 (p=0.011) and CD28 (p=0.026) (figure 5E). Two significantly enriched pathways were observed in the low expression cohort. Detailed enrichment profiles and related genes were also depicted (online supplemental figure 4).

10.1136/jitc-2021-002339.supp5 Supplementary data

Besides, based on CC for GSEA analysis, genes negatively correlated with FOXP3 were also significantly enriched in another three pathways ‘KEGG_PARKINSONS_DISEASE’, ‘KEGG_HUNTINGTONS_DISEASE’, and ‘KEGG_OXIDATIVE_PHOSPHORYLATION’ (online supplemental figure 5). Among all overlapped genes that presented in more than two pathways, expression of cytochrome c oxidase subunit 8A (*COX8A*) suggested the highest negative correlation with FOXP3 level, indicating it might play an important role in the regulation of FOXP3 (CC=−0.648, p=0.001; online supplemental figure 5). For genes positively correlated with FOXP3, overlapped genes among the top four pathways only presented low to moderate correlations with FOXP3 (online supplemental figure 6).

10.1136/jitc-2021-002339.supp6 Supplementary data

10.1136/jitc-2021-002339.supp7 Supplementary data

### Tumor-infiltrating immune profiles in patients with SCLC based on immune risk score

**Figure 6:**
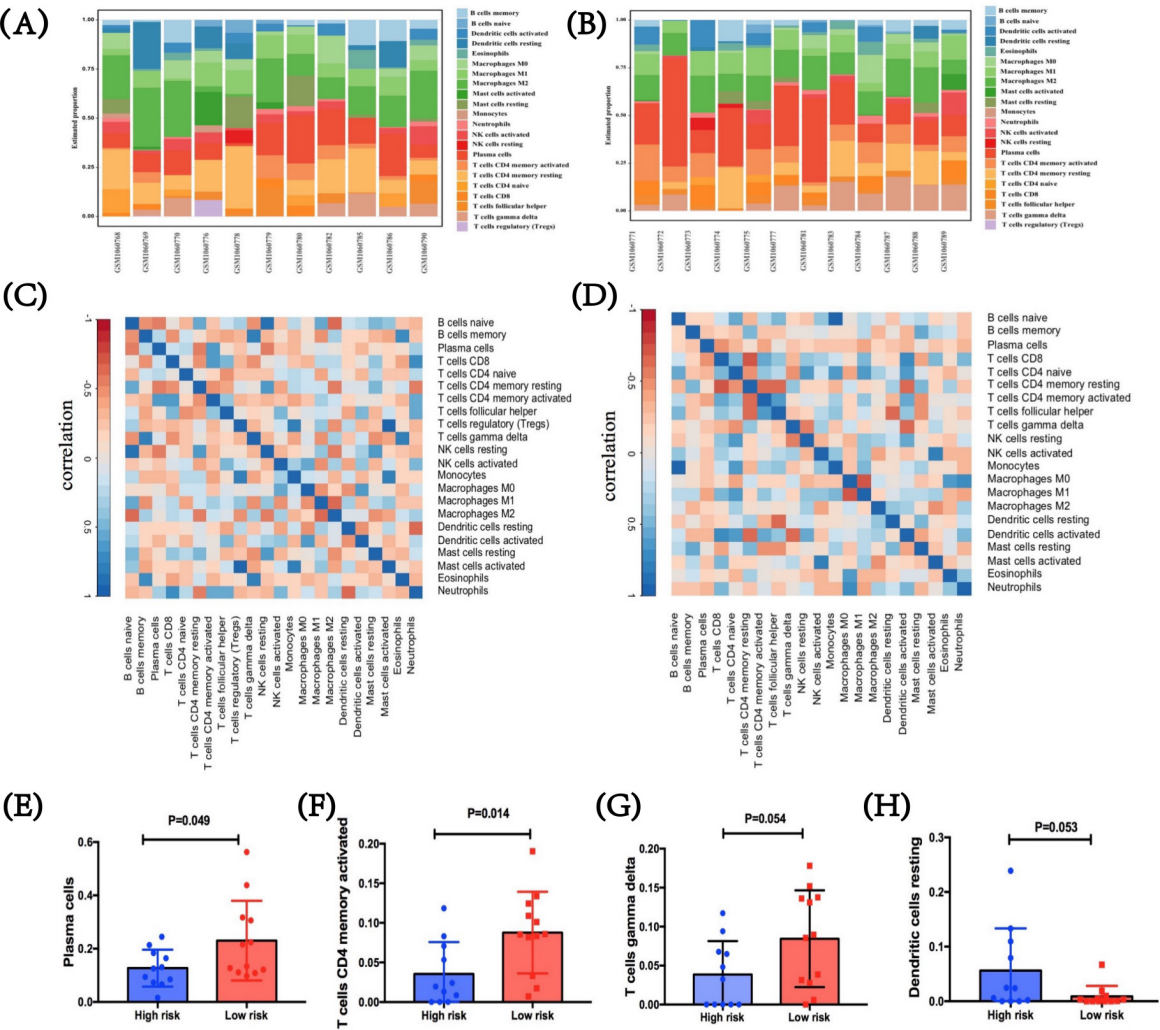
Tumor-infiltrating immune profiles in patients with SCLC based on immune risk score. (A, B) Immune features between the high-immune risk group and the low-immune risk group. Two heatmaps showed the difference in the proportion of diverse 22 immune cells in diverse samples. (C, D) The correlations among these immune cells in TME in both high-immune risk and low-immune risk groups, respectively, and the interactions vary between different cells. (E–H) Immune-cell proportions between high-immune risk and low-immune risk groups. SCLC, small-cell lung cancer; TME, tumor microenvironment.

To figure out tumor-infiltrating condition of patients with SCLC in two immune risk groups, we further performed CIBERSORT using LM22.^26^ In the SCLC GEO dataset, two heatmaps were first performed with immune features between these two immune risk groups of high or low level, which showed the difference in the proportion of 22 diverse immune cells in different samples (figure 6A, B). After analysis, we summarized the correlations among these immune cells in TME, respectively, in both groups. The interactions vary between different cells. For the high-immune risk group, Treg cells indicated a strong correlation with monocyte (CC=0.820, p=0.002) and activated mast cells (CC=0.980, p<0.001), while a relatively weak correlation was shown between naïve B cells and neutrophils (CC=−0.326, p=0.328; figure 6C). For the low-immune risk group, naïve B cells showed an extremely high correlation with monocytes (CC=0.942, p<0.001), while its relationship with other cell types was quite modest (figure 6D). Further, immune-cell proportions were explored between high-immune and low-immune risk groups (figure 6E–H). By incorporating the expression levels of the three predictive biomarkers, significant differences were found in activated CD4^+^ memory T cells (p=0.014) and plasma cells (p=0.049), and marginally marked differences in gamma delta T cells (γδ T cells) (p=0.054) and resting dendritic cells (p=0.053). Specifically, three immune cells including activated CD4^+^ memory T cells, γδ T cells, and plasma cells were higher in the low-immune risk group compared with those in the high-score group. Conversely, in comparison with low-risk group, resting dendritic cell level was suggested higher in the high-immune risk group. Therefore, the heterogeneity of tumor-infiltrating immune cells might represent a promising feature for risk prediction in SCLC. In return, these meaning findings also validated the crucial role of immune risk scores in TME.

## Discussion

In this study, we appraised the expression status of FOXP3 on TILs at first. All clinical factors excluding SCLC clinical staging showed no significant impacts on FOXP3 expression status on TILs. Then, we found that status of FOXP3 expression was closely associated with the expression of other immune molecules, and relapse time of patients with SCLC. More importantly, compared with FOXP3-positive group, negative FOXP3 expression on TILs predicted earlier recurrence of patients with SCLC. Therefore, FOXP3-based immune risk scoring system and nomogram model were constructed with good prognostic ability. Most results were verified by public database. After downloading suitable datasets from GEO, we found the statistically different expression of FOXP3, which inspired us to further study FOXP3-related biological pathways and regulatory genes in SCLC. In addition, we outlined the immune landscapes remodeled by the FOXP3-based immune risk score system and revealed the heterogeneity of tumor-infiltrating immune cells in SCLC samples.

FOXP3 is an important member of the forkhead/winged-helix family of transcription regulators. However, as a transcription factor, FOXP3 may repress transcription when activated.^28^ Human CD8^+^ and CD4^+^ T cells may also upregulate FOXP3 and obtain inhibited properties after activation.^29–31^ Colombo and Piconese reviewed that local Treg number in TME was closely correlated with tumor progression and prognosis.^32^ According to previous studies, Tregs could suppress the activation and differentiation of CD8^+^ cytotoxic T cells as well as CD4^+^ helper T cells, which could induce reactivity against tumor-expressed antigens.^33–35^ Beyond correlating with these markers, FOXP3 also has interactions with checkpoints in tumors. In glioblastoma, immunosuppression could be promoted by upregulating PD-L1 and Tregs, which indicated PD-L1 might expand immunosuppressive Tregs.^36^ These studies suggested FOXP3 expression of immunocytes played crucial roles in regulating tumor immunity. The expression of FOXP3 protein has been determined in various cancer types, such as breast, NSCLC, glioblastoma, and colorectal cancers.^9 10 36 37^ However, few data were available for SCLC. Thus, we first focused on FOXP3 expression level on TILs and its interactions with other biomarkers.

In our study, we discovered that FOXP3 status was statistically associated with immune checkpoints, including PD-1 and PD-L1 expressed on TILs. We also found the coexpression of FOXP3 and immune markers, including CD3, CD4, and CD8 in our data. However, the correlations of FOXP3 with PD-L1 or CD8 were not revealed from public dataset correspondingly. The following reasons might explain this inconsistency. First, only nine SCLC cases were available forming the external cohort. The sample size was extremely small for analysis. Second, variability between study designs may also affect results. Methods for expression detection were different. The expression profiles were obtained via RNA-seq in the public dataset, but not IHC. Thus, prospective studies with large populations are urgently required. Bioinformatics analysis of the cBioportal dataset also showed the significant correlation of FOXP3 level with LAG-3 expression in SCLC. From the aforementioned analysis, we can find the extensive interaction of FOXP3 with other immune biomarkers in SCLC. In TME, Tregs were found to upregulate inhibitory immune checkpoints, which could indirectly suppress the activation of effector T cells through influencing APC functions negatively.^18 38^ In addition to these immune biomarkers we analyzed, the relationship of FOXP3 with other proteins, such as CD25, CD39, TIM-3, CTLA-4, and TIGIT, was also explored and found upregulated in CD4^+^FOXP3^+^ Tregs.^39–41^ Currently, the biological significance of the simultaneous overexpression of immune checkpoints and activation biomarkers in tumor-infiltrating Tregs remains unclear, which should be further evaluated in the context of immune checkpoint inhibitor-based immunotherapy. Full exploration of mechanisms of FOXP3 interacting with other immune biomarkers in TME is warranted.

By survival analysis, we found that patients with SCLC with positive FOXP3 levels had longer RFS when compared with the negative group. There are many conflicting results on the clinical value of FOXP3 with regard to prognostic prediction in certain malignancies. Studies found that the increased frequency of FOXP3-positive Tregs in tumors had relationship with improved prognosis in certain cancer types, such as colorectal carcinoma^13 16 42^ as well as head and neck cancer.^14^ A previous study proposed that infiltration of FOXP3-positive Tregs helped to suppress inflammatory response of gut microbes in colorectal cancer at early stages.^43^ However, meta-analysis including 74 studies that encompassed 17 cancer types (15 512 patients) indicated that high FOXP3-positive Treg infiltration in tumors was correlated with poor prognosis in most solid malignancies studied.^17^ Many reasons might cause these contradictory results. First, the prognostic value of the FOXP3 level on TILs on clinical outcomes can vary with tumor types, histological grade, as well as molecular subtype. Besides, different FOXP3 proteins may have distinct functions, which can affect their prognostic effect. Moreover, the sample size, study design, cut-off values, and technology used can also contribute to the difference. FOXP3 is considered as an important factor through tumor development. By in vitro assays and in vivo tumor xenograft method, Yang and colleagues found that FOXP3 might act to promote the formation of β-catenin–TCF4 complex, which could facilitate the activation of epithelial–mesenchymal transition related molecules, such as slug and snail, leading to growth and metastasis in NSCLC.^37^ By contrast, in colorectal cancers, TILs with positive FOXP3 status presented heterogeneously in both non-suppressive and suppressive forms, whose impacts on prognosis remained controversial.^16 44 45^ When FOXP3^+^ Tregs emerged as good citizens, they may mainly be responsible for inhibiting ‘tumor promoting inflammation’ in chronic inflammatory infiltrates. Further exploring the potential mechanisms of FOXP3 is important to better understand its capability in SCLC. We suggested to carefully balance the suppressive and non-suppressive roles when considering FOXP3 as a target against cancer.

Subgroup analysis showed that on TILs, positive FOXP3 protein combining with immune checkpoint PD-1, PD-L1, or LAG-3 positive was statistically associated with longer RFS. The favorable outcomes were also found when positive FOXP3 protein in combination with CD3, CD4, or CD8 positive on TILs. In SCLC, higher CD3 expression was considered associated with better prognosis, while no or even contrary influence was shown in the condition of PD-L1 overexpression.^46 47^ Conversely, patients with SCLC with higher expression of PD-L1 and CD8 were found associated with better survival in the study of Sun *et al*.^48^ Additionally, Bonanno and colleagues found that FOXP3 expression had prognostic value for OS in non-metastatic SCLC.^49^ FOXP3^+^/CD8^+^ T cell ratio was supposed as a negative prediction for patients’ prognosis in many cancers. However, few studies analyzed the clinical significance and prognostic value when FOXP3 combined with other immune markers. Our study might fill the gaps and demonstrated the possible prognostic value of FOXP3 in combination with checkpoints and immune markers in this filed.

Given the extensive interaction and the meaningful subgroup analysis results of FOXP3 with other immune biomarkers, we put forward the use of XGBoost machine learning for the importance evaluation of these markers and selected the top three markers for construction of a combined biomarker. In comparison with single biomarker, this FOXP3-based immune risk score model showed better prediction performance. Besides, when comparing the prediction performance between this XGBoost-based model and risk systems constructed on the basis of FOXP3 and TIL PD-L1, TIL PD-1, TIL LAG-3, CD3, CD4, CD8, or PD-L1 on tumor cells, we found this XGBoost-based model exhibited the best prediction performance with the maximal AUC value. Based on this, we further constructed a nomogram model which provided a personalized system for patients with SCLC to predict recurrence. Good performance was determined in both training set and validation set. When integrating immune risk score and SCLC staging to predict the probability of RFS of 1, 3, and 5 years, all sets including the entire set, training set, and validation set showed that low-risk groups were correlated with longer RFS compared with moderate-risk and high-risk groups. However, no significant difference was obtained in the validation cohort (p=0.126). Further pairwise comparison between two risk groups was conducted. Marginal significance was observed in the comparison between low-risk and high-risk groups (p=0.050), while no significant survival difference between moderate risk and low or high risk groups (p=0.644 and p=0.133, respectively). This result might be ascribed to the limited sample size in the validation cohort (n=31). Besides, the median follow-up time was only 39.400 months in our study. Longer follow-up time would better evaluate the prognosis of patients with SCLC. Given all of these, the clinical value of the FOXP3-based nomogram in SCLC remains to be further elucidated in future studies.

Based on public datasets, the clinical values of FOXP3 expression and immune risk score were verified. However, the difference between the low-risk/moderate-risk groups and the high-risk group showed no statistical significance, which may be caused by several factors. First, the sample size of patients with SCLC who met the criteria was only nine, so it failed to evaluate predictive ability and clinical applications of FOXP3 in SCLC objectively. Besides, diverse clinical end points may also lead to this difference. Study design and technology used may also affect the results. Further studies are needed to elucidate the risk model in SCLC.

The results of the GSEA showed that the top three high-FOXP3-related enrichment pathways were intestinal immune network for IgA production and immune diseases including primary immunodeficiency and allograft rejection. *CD28*, *CD40LG*, *HLA-DMA*, and *IL-2*, were significantly higher-expressed in high FOXP3 expression group when compared with low expression, in which CD28 and IL-2 were statistically correlated with FOXP3. The important roles of CD28 and IL-2 in the regulation of FOXP3 expression have been well investigated by previous studies. CD28, the ligand of B7-1 (CD80) and B7-2 (CD86), is the major costimulatory molecule on T lymphocytes.^50^ Tai and colleagues suggested that CD28 costimulation could directly signal developing thymocytes to express FOXP3.^50^ IL-2 signaling is a key cytokine for the activation, proliferation, and differentiation of T cells.^51^ The expression of IL-2 α‐chain (CD25) is induced by CD28 and T-cell receptor (TCR) signals and forms high-affinity IL-2R, together with the common-chain (CD132) and IL-2R β ‐chain (CD122).^52 53^ In the presence of TCR and transforming growth factor‐β signaling, activation of FOXP3 transcription is enhanced by IL-2 signaling.^54^ The relative regulation is summarized in online supplemental figure 7. Besides, in the low FOXP3 expression-related enrichment pathways, we also revealed FOXP3 had significant correlation with the overlapped genes *MUTYH*, *POLD1*, and *POLD2*, which belonged to basic excision repair (BER) pathway. Regrettably, current research on FOXP3 and DNA damage response and repair (DDR) mainly involves mismatch repair,^55 56^ and few research is available on BER. At present, DDR deficiency has been studied related to high-tumor mutation burden and activated anti-tumor immunity.^57 58^ TME alteration may affect the regulation of FOXP3 expression. The specific relationship between these genes and FOXP3 needs to be further explored.

10.1136/jitc-2021-002339.supp8 Supplementary data

Our study revealed that genes negatively correlated with FOXP3 were enriched in pathways such as neurodegenerative disease-related pathways and oxidative phosphorylation pathway. Few reports suggested the relationship between FOXP3 expression with Parkinson and Huntington diseases. As for oxidative phosphorylation, there are different opinions about its relationship with FOXP3. It was indicated that FOXP3 deficiency could cause dysregulation of metabolic checkpoint kinase mammalian target of rapamycin complex 2 signal and enhance oxidative phosphorylation.^59^ Also, recently, Zappasodi and colleagues found that blocking oxidative phosphorylation could downregulate FOXP3 expression.^60^ Therefore, further studies are required for the relationship between FOXP3 and oxidative phosphorylation. Besides, among all overlapped genes presented in more than two gene sets, we found that *COX8A*, which encoded protein of the terminal enzyme of respiratory chain, showed the highest negative correlation with FOXP3 level, indicating it might play an important role in regulating FOXP3. At present, few data showed their relationship. The meaningful finding of the relationship between *COX8A* and *FOXP3* is worth for further deep exploration. GSEA also revealed that FOXP3 had an extensively positive association with other molecules in TME, such as CD44, CR1, IL1R, and CD36. In addition, epigenetic mechanisms also contribute to the transcriptional regulation of FOXP3.^61 62^ It was found that acetyltransferases, deacetylases, and kinases that could regulate post-translational modifications of FOXP3 were potential targets for regulating FOXP3 activity.^63^ For exploration of the TME traits, activated CD4^+^ memory T cells, γδ T cells, and plasma cells were analyzed higher in the low-immune risk group compared with those in the high-score group. These findings illustrated the heterogeneity of tumor-infiltrating immune cells in different groups. Therefore, FOXP3 and its regulators are worth further exploring as potential therapeutic targets. The correlation of FOXP3 and immune checkpoints may inspire the development of combination strategies.

This study had some limitations. First, this was a retrospective as well as single-centered study. Besides, our hypothesis and results were based on a small sample size. Prospective and multicentered studies are required in the future.

## Conclusion

In conclusion, we investigated the expression patterns of FOXP3 in SCLC and revealed the close interaction between FOXP3 and other immune biomarkers in TME from proteomic and transcriptomic levels. Meanwhile, our work highlighted the crucial prognostic value of FOXP3 in predicting relapse of SCLC at stages I–III, constructed an immune risk score system, and explored FOXP3-based clinical prediction model by integrating immune risk score and SCLC staging. We also discovered the important effects of CD28 and IL-2 signaling in regulating FOXP3 expression and proposed that BER and oxidative phosphorylation might relate to FOXP3 regulation. The immune landscape in each immune risk group was further depicted, revealing the heterogeneity of tumor-infiltrating immune cells. Further investigation was required for investigation and validation of the regulation mechanisms of FOXP3 so as to better understand its effects on TME and promote antitumor therapy.

## Data Availability

Data are available upon reasonable request. The data used to support the findings of this study are available from the corresponding author upon request.
