# Supplementary material for: FOXP3-based immune risk model for recurrence prediction in small-cell lung cancer at stages I–III
Source: J Immunother Cancer. 2021 May 18;9(5):e002339. doi: 10.1136/jitc-2021-002339 (PMC8137193; doi:10.1136/jitc-2021-002339)
Supplement: Supplementary data [file jitc-2021-002339supp001.pdf]

BMJ Publishing Group Limited (BMJ) disclaims all liability and responsibility arising from any reliance placed on this supplemental material which has been supplied by the author(s)

Online supplemental tables: Table S1-S8

Table S1. Data of clinical characteristics and FOXP3 expression level in SCLC patients enrolled in this study.

							Pathological review		FOXP3 expression on TILs		
**Patient ID**	**Sex**	**Age, year**	**Smoke**	**Stage**	**RFS (months)**	**RFS status**	**Pathological review 1**	**Pathological review 2**	**review 1**	**review 2**	**review 1+2**
1	Male	55	Non-smoker	I	63	1	SCLC	SCLC	positive	positive	positive
2	Male	52	Non-smoker	III	1.2	1	SCLC	SCLC	negative	negative	negative
3	Male	76	Non-smoker	I	5	1	SCLC	SCLC	positive	positive	positive
4	Male	58	Non-smoker	II	69.4	0	SCLC	SCLC	positive	positive	positive
5	Male	55	Non-smoker	I	66.3	0	SCLC	SCLC	positive	positive	positive
6	Male	51	Non-smoker	III	6	1	SCLC	SCLC	negative	negative	negative
7	Male	76	Smoker	III	6	1	SCLC	SCLC	positive	positive	positive
8	Female	47	Non-smoker	III	65.7	0	SCLC	SCLC	positive	positive	positive
9	Female	65	Non-smoker	III	62.2	0	SCLC	SCLC	positive	positive	positive
10	Male	72	Non-smoker	III	14.5	1	SCLC	SCLC	negative	negative	negative
11	Male	73	Non-smoker	III	45	1	SCLC	SCLC	positive	positive	positive
12	Male	56	Non-smoker	III	15.6	1	SCLC	SCLC	negative	negative	negative
13	Male	66	Smoker	III	32	1	SCLC	SCLC	positive	positive	positive
14	Male	58	Smoker	III	1	1	SCLC	SCLC	positive	negative	positive

Jiang M, *et al. J Immunother Cancer* 2021; 9:e002339. doi: 10.1136/jitc-2021-002339

15	Female	68	Non-smoker	III	2	1	SCLC	SCLC	positive	positive	positive
16	Male	60	Non-smoker	II	54	0	SCLC	SCLC	positive	positive	positive
17	Male	67	Non-smoker	II	54.1	0	SCLC	SCLC	positive	positive	positive
18	Female	43	Non-smoker	II	49.1	0	SCLC	SCLC	positive	positive	positive
19	Male	76	Non-smoker	III	1.2	1	SCLC	SCLC	negative	negative	negative
20	Male	53	Non-smoker	I	1	1	SCLC	SCLC	positive	positive	positive
21	Male	60	Smoker	III	41.3	1	SCLC	SCLC	positive	positive	positive
22	Female	51	Non-smoker	III	8.2	1	SCLC	SCLC	negative	negative	negative
23	Male	52	Non-smoker	III	15.2	1	SCLC	SCLC	negative	negative	negative
24	Male	78	Non-smoker	I	49	0	SCLC	SCLC	positive	positive	positive
25	Male	54	Non-smoker	III	5.2	1	SCLC	SCLC	positive	positive	positive
26	Male	74	Non-smoker	III	5	1	SCLC	SCLC	positive	positive	positive
27	Male	60	Non-smoker	II	48	0	SCLC	SCLC	positive	positive	positive
28	Female	68	Non-smoker	III	38.1	1	SCLC	SCLC	positive	positive	positive
29	Female	66	Non-smoker	II	48.3	0	SCLC	SCLC	positive	positive	positive
30	Male	73	Non-smoker	I	18	1	SCLC	SCLC	negative	positive	positive
31	Male	65	Non-smoker	II	48	0	SCLC	SCLC	positive	positive	positive
32	Male	67	Smoker	I	1.7	1	SCLC	SCLC	positive	positive	positive
33	Female	73	Non-smoker	III	4.8	1	SCLC	SCLC	positive	positive	positive

Jiang M, *et al. J Immunother Cancer* 2021; 9:e002339. doi: 10.1136/jitc-2021-002339

34	Male	75	Smoker	I	41.2	1	SCLC	SCLC	positive	positive	positive
35	Male	59	Smoker	II	45.3	0	SCLC	SCLC	positive	positive	positive
36	Male	75	Smoker	I	17	1	SCLC	SCLC	positive	positive	positive
37	Male	65	Smoker	I	8.5	1	SCLC	SCLC	positive	positive	positive
38	Female	63	Non-smoker	I	41	0	SCLC	SCLC	positive	positive	positive
39	Male	57	Smoker	III	1	1	SCLC	SCLC	positive	positive	positive
40	Male	40	Non-smoker	I	6.3	1	SCLC	SCLC	positive	positive	positive
41	Male	42	Smoker	II	1	1	SCLC	SCLC	positive	positive	positive
42	Male	58	Non-smoker	II	15	1	SCLC	SCLC	negative	negative	negative
43	Male	54	Smoker	III	41	0	SCLC	SCLC	positive	positive	positive
44	Male	72	Smoker	III	6	1	SCLC	SCLC	positive	positive	positive
45	Female	56	Non-smoker	II	39.4	0	SCLC	SCLC	negative	negative	negative
46	Male	66	Smoker	I	8.3	1	SCLC	SCLC	positive	positive	positive
47	Male	70	Smoker	III	11	1	SCLC	SCLC	negative	negative	negative
48	Male	52	Smoker	III	37	0	SCLC	SCLC	positive	positive	positive
49	Male	66	Smoker	III	2	1	SCLC	SCLC	positive	positive	positive
50	Male	77	Smoker	III	36	0	SCLC	SCLC	positive	positive	positive
51	Female	63	Non-smoker	III	14	1	SCLC	SCLC	negative	negative	negative
52	Male	74	Smoker	III	18	1	SCLC	SCLC	positive	positive	positive

53	Male	64	Smoker	II	37.1	0	SCLC	SCLC	positive	positive	positive
54	Male	67	Non-smoker	III	36	0	SCLC	SCLC	positive	positive	positive
55	Male	81	Smoker	I	12.2	1	SCLC	SCLC	positive	positive	positive
56	Male	38	Non-smoker	I	35.5	0	SCLC	SCLC	positive	positive	positive
57	Male	63	Smoker	II	35.2	0	SCLC	SCLC	positive	positive	positive
58	Male	76	Non-smoker	III	7	1	SCLC	SCLC	positive	positive	positive
59	Male	67	Smoker	III	20.3	1	SCLC	SCLC	positive	positive	positive
60	Male	74	Smoker	III	14.7	1	SCLC	SCLC	negative	negative	negative
61	Female	67	Non-smoker	I	1.6	1	SCLC	SCLC	positive	positive	positive
62	Male	63	Non-smoker	III	16.2	1	SCLC	SCLC	positive	positive	positive
63	Female	47	Non-smoker	II	1	1	SCLC	SCLC	positive	positive	positive
64	Male	66	Smoker	II	6	1	SCLC	SCLC	positive	positive	positive
65	Male	68	Non-smoker	I	33.4	0	SCLC	SCLC	positive	positive	positive
66	Male	61	Non-smoker	I	33	0	SCLC	SCLC	positive	positive	positive
67	Male	72	Smoker	I	31	0	SCLC	SCLC	positive	positive	positive
68	Male	68	Smoker	I	10	1	SCLC	SCLC	positive	positive	positive
69	Female	61	Non-smoker	I	29.2	0	SCLC	SCLC	positive	positive	positive
70	Male	49	Smoker	III	31.4	0	SCLC	SCLC	positive	positive	positive
71	Male	68	Non-smoker	III	28.5	1	SCLC	SCLC	positive	positive	positive

72	Male	54	Non-smoker	II	31	0	SCLC	SCLC	positive	positive	positive
73	Male	52	Non-smoker	I	31.6	0	SCLC	SCLC	positive	positive	positive
74	Male	58	Smoker	III	31	0	SCLC	SCLC	positive	positive	positive
75	Male	63	Smoker	III	29.3	0	SCLC	SCLC	positive	positive	positive
76	Male	64	Smoker	II	6	1	SCLC	SCLC	positive	positive	positive
77	Male	51	Smoker	II	15	1	SCLC	SCLC	positive	positive	positive
78	Male	61	Smoker	I	28	0	SCLC	SCLC	positive	positive	positive
79	Male	68	Non-smoker	III	28.2	0	SCLC	SCLC	positive	positive	positive
80	Male	62	Smoker	II	1	1	SCLC	SCLC	positive	positive	positive
81	Female	63	Non-smoker	III	25.2	0	SCLC	SCLC	positive	positive	positive
82	Male	55	Non-smoker	III	10	1	SCLC	SCLC	positive	positive	positive
83	Male	66	Smoker	I	10.5	1	SCLC	SCLC	positive	positive	positive
84	Male	73	Smoker	II	1	1	SCLC	SCLC	positive	positive	positive
85	Male	63	Non-smoker	I	21.6	0	SCLC	SCLC	positive	positive	positive
86	Male	65	Non-smoker	I	21	0	SCLC	SCLC	positive	positive	positive
87	Male	69	Smoker	I	20	0	SCLC	SCLC	positive	positive	positive
88	Male	42	Non-smoker	III	9	1	SCLC	SCLC	positive	positive	positive
89	Female	70	Non-smoker	I	19	0	SCLC	SCLC	positive	positive	positive
90	Male	51	Non-smoker	I	19.7	0	SCLC	SCLC	positive	positive	positive

Jiang M, *et al. J Immunother Cancer* 2021; 9:e002339. doi: 10.1136/jitc-2021-002339

91	Male	60	Non-smoker	II	19	0	SCLC	SCLC	positive	positive	positive
92	Male	68	Smoker	III	12.1	1	SCLC	SCLC	positive	positive	positive
93	Male	66	Smoker	I	19	0	SCLC	SCLC	positive	positive	positive
94	Male	72	Smoker	I	18.3	0	SCLC	SCLC	negative	negative	negative
95	Male	67	Smoker	I	18.2	0	SCLC	SCLC	positive	positive	positive
96	Female	63	Non-smoker	I	18	0	SCLC	SCLC	positive	positive	positive
97	Male	63	Non-smoker	II	11.3	1	SCLC	SCLC	negative	negative	negative
98	Male	78	Smoker	I	17	0	SCLC	SCLC	positive	positive	positive
99	Male	51	Smoker	I	3.2	1	SCLC	SCLC	positive	positive	positive
100	Male	69	Smoker	I	6	1	SCLC	SCLC	positive	positive	positive
101	Male	62	Non-smoker	I	17	0	SCLC	SCLC	positive	positive	positive
102	Female	61	Non-smoker	I	17.9	0	SCLC	SCLC	positive	positive	positive

Table S2. Characteristics of the 102 patients

Characteristics	Number, n (%)
Age, years	
<65	53, (52.0)
≧65	49, (48.0)
Gender, n (%)	
Male	84, (82.4)
Female	18, (17.6)
Smoking status, n (%)	
Smoker	44, (43.1)
Non-smoker	58, (56.9)
T stage, n (%)	
1	40, (39.2)
2	47, (46.1)
3	13, (12.7)
4	2, (2.0)
N stage	
0	44, (43.1)

Jiang M, *et al. J Immunother Cancer* 2021; 9:e002339. doi: 10.1136/jitc-2021-002339

1	23, (22.5)
2	34, (33.3)
3	1, (0.1)
M stage	
0	98, (96.1)
1	4, (3.9)
Stage	
I	38, (37.3)
II	22, (21.6)
III	42, (41.2)
IV	0, (0)
Chemotherapy	
Yes	54, (52.9)
No	48, (47.1)

Table S3. Correlation between FOXP3 expression on TILs and clinical data

	FOXP3 expression on TILs (total: 102)		
	**Positive, N (%)**	**Negative, N (%)**	**P value**
Age, median			
<65	44, (43.1)	9, (8.9)	0.320
≧65	44, (43.1)	5, (4.9)	
Gender			
Male	73, (71.6)	11, (10.8)	0.689
Female	15, (14.7)	3, (2.9)	
Smoking status			
Smoker	41, (40.2)	3, (2.9)	0.077
Non-smoker	47, (46.1)	11, (10.8)	
Stage			
I- II	56, (54.9)	4, (3.9)	**0.013**
III	32, (31.4)	10, (9.8)	
Metastasis			
Yes	3, (2.9)	1, (1.0)	0.504
No	85, (83.3)	13, (12.7)	

Chemotherapy			
Yes	45, (44.1)	9, (8.8)	0.360
No	43, (42.2)	5, (4.9)	

Table S4. Relationship between the expression level of FOXP3 and other immune biomarkers

	FOXP3 on TILs	
Biomarkers/parameter	Correlation coefficient	P value
PD-L1 on tumor cells	0.056	0.573
PD-1 on TILs	0.327	**0.001**
PD-L1 on TILs	0.307	**0.002**
LAG-3 on TILs	0.204	**0.040**
CD3 on TILs	0.366	**0.000**
CD4 on TILs	0.307	**0.002**
CD8 on TILs	0.215	**0.030**

Table S5. Univariate and multivariate analysis for prediction of FOXP3 expression on TILs in SCLC patients

Variables	Univariate			Multivariate		
	Odds ratio	95% CI	P value	Odds ratio	95% CI	P value
Age (<65 y *vs*. ≥65 y)	0.556	0.172-1.791	0.325			
Sex (female *vs*. male)	0.753	0.187-3.032	0.690			
Smoking status (nonsmoker *vs*. smoker)	0.313	0.082-1.198	0.090			
Stage (I–II *vs*. III)	4.375	1.268-15.091	**0.019**	4.381	1.168-16.428	**0.028**
PD-L1 on tumor cells (negative *vs*. positive)	0.000	Undefined	0.999			
PD-1 on TILs (negative *vs*. positive)	0.000	Undefined	0.997			

PD-L1 on TILs (negative *vs*. positive)	0.000	Undefined	0.998			
LAG-3 on TILs (negative *vs*. positive)	0.219	0.046-1.039	0.056			
CD3 on TILs (negative *vs*. positive)	0.051	0.006-0.406	**0.005**	0.051	0.006-0.415	**0.005**
CD4 on TILs (negative *vs*. positive)	0.000	Undefined	0.998			
CD8 on TILs (negative *vs*. positive)	0.135	0.017-1.077	0.059			

Table S6. Correlation of clinical factors and RFS in SCLC

Characteristic	Log-rank (Mantel-Cox)		Median survival	
	Chi square	P-value	value	95% CI
Age, n%	3.432	0.064	32.000	12.106-51.894
<65			63.000	1.985-124.015
≥65			18.000	10.666-25.334
Gender	1.685	0.194	32.000	12.106-51.894
Female			Undefined	Undefined
Male			18.000	2.106-33.894
Smoking status	3.852	0.050	32.000	12.106-51.894
Non-smoker			45.000	18.814-71.186
Smoker			15.000	6.645-23.355
Stage	8.141	**0.004**	32.000	12.106-51.894
Stage I–II			63.000	26.271-99.729
Stage III			14.700	10.360-19.040
FOXP3 on TILs	7.093	**0.008**	32.000	12.106-51.894
Negative			14.000	8.133-19.867
Positive			41.200	26.937-55.463
PD-L1 on TILs	8.137	**0.004**	32.000	12.106-51.894

Jiang M, *et al. J Immunother Cancer* 2021; 9:e002339. doi: 10.1136/jitc-2021-002339

Negative			14.700	10.192-19.208
Positive			41.300	41.076-41.524
LAG-3 on TILs	3.022	0.082	32.000	12.106-51.894
Negative			15.000	0.000-31.011
Positive			41.200	25.001-57.399
CD3 on TILs	7.669	**0.006**	32.000	12.106-51.894
Negative			14.000	9.150-18.850
Positive			41.300	28.503-54.097
CD4 on TILs	7.136	**0.008**	32.000	12.106-51.894
Negative			15.000	11.082-18.918
Positive			Undefined	Undefined
CD8 on TILs	7.998	**0.005**	32.000	12.106-51.894
Negative			15.000	10.353-19.647
Positive			Undefined	Undefined

Table S7. Clinical characteristics of patients as validation set from cBioportal

**Characteristics**	**Number, n (%)**
Age, years	
<65	2, (22.2)
≧65	7, (77.8)
Gender, n (%)	
Male	6, (66.7)
Female	3, (33.3)
Smoking status, n (%)	
Smoker	7, (77.8)
Non-smoker	1, (11.1)
Unknown	1, (11.1)
T stage, n (%)	
1	6, (66.7)
2	2, (22.2)
3	1, (11.1)
N stage	
0	6, (66.7)

1	1, (11.1)
2	1, (11.1)
3	1, (11.1)
M stage	
0	8, (88.9)
1	1, (11.1)
Stage	
I	6, (66.7)
II	0, (0.0)
III	2, (22.2)
IV	1, (11.1)
Chemotherapy	
Yes	4, (44.5)
No	2, (22.2)
Unknown	3, (33.3)

Jiang M, *et al. J Immunother Cancer* 2021; 9:e002339. doi: 10.1136/jitc-2021-002339

Table S8. Relationship between the expression level of FOXP3 and clinicalpathological factors in the cbioportal database

	**FOXP3** expression in SCLC	
Biomarkers/parameter	Correlation coefficient	P value
PD-L1 expression	0.212	0.584
PD-1 expression	0.894	**0.001**
LAG-3 expression	0.775	**0.014**
CD3	0.883	**0.002**
CD4	0.773	**0.015**
CD8	0.011	0.977
Diagnosis age	0.466	0.206
Gender	0.414	0.379

Jiang M, *et al. J Immunother Cancer* 2021; 9:e002339. doi: 10.1136/jitc-2021-002339
