# Supplementary figures and images for: FOXP3-based immune risk model for recurrence prediction in small-cell lung cancer at stages I–III
Source: J Immunother Cancer. 2021 May 18;9(5):e002339. doi: 10.1136/jitc-2021-002339 (PMC8137193; doi:10.1136/jitc-2021-002339)

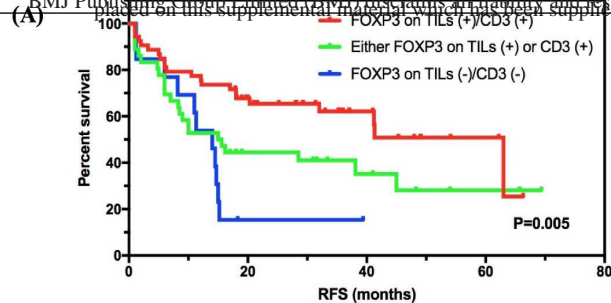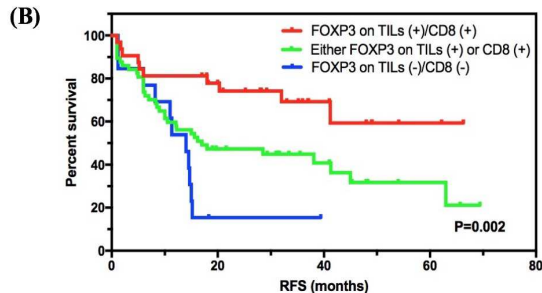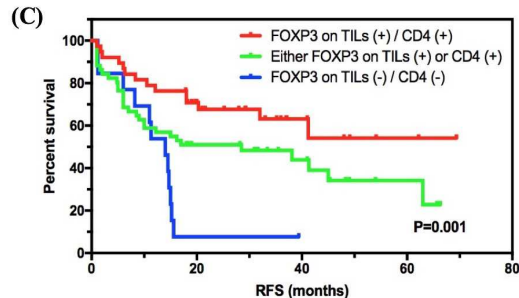

Supplement: Supplementary data [file jitc-2021-002339supp002.pdf]

(A)

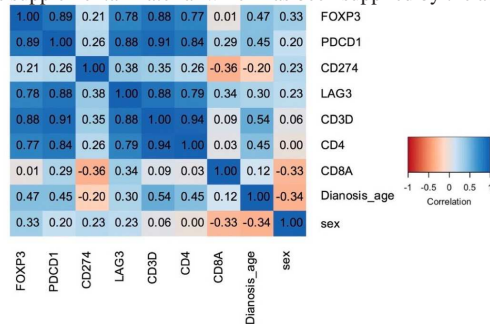

(B)

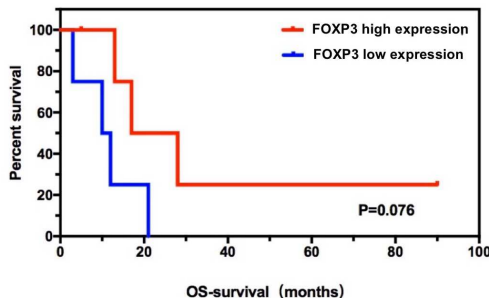

(C)

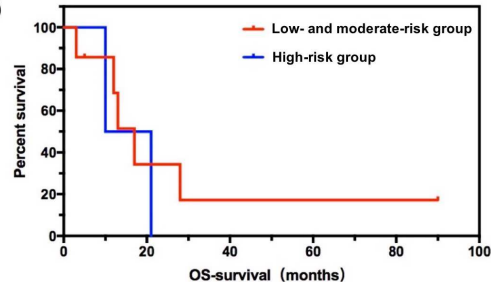

Supplement: Supplementary data [file jitc-2021-002339supp003.pdf]

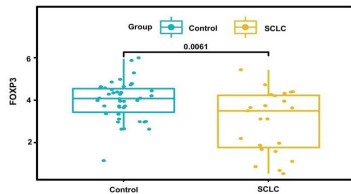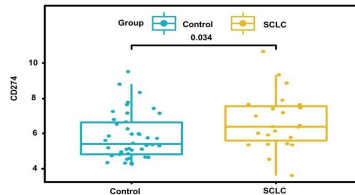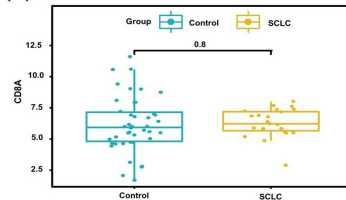

Supplement: Supplementary data [file jitc-2021-002339supp004.pdf]

(A)

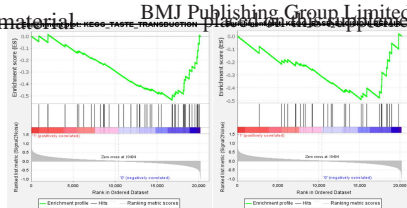

(B)

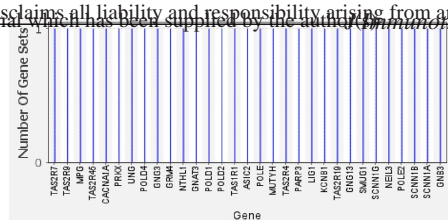

(C)

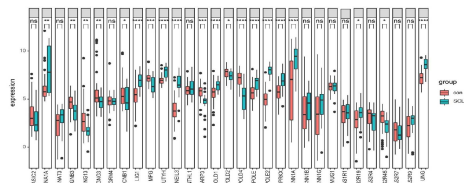

(D)

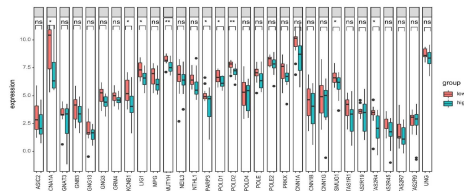

(E)

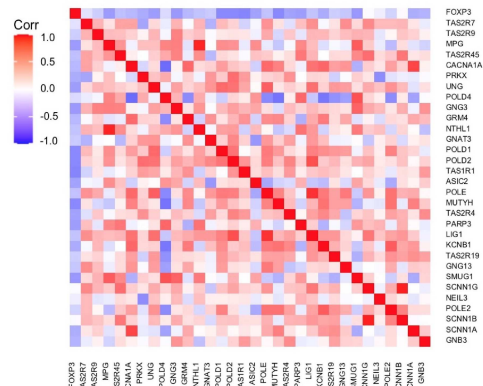

Supplement: Supplementary data [file jitc-2021-002339supp005.pdf]

(A)

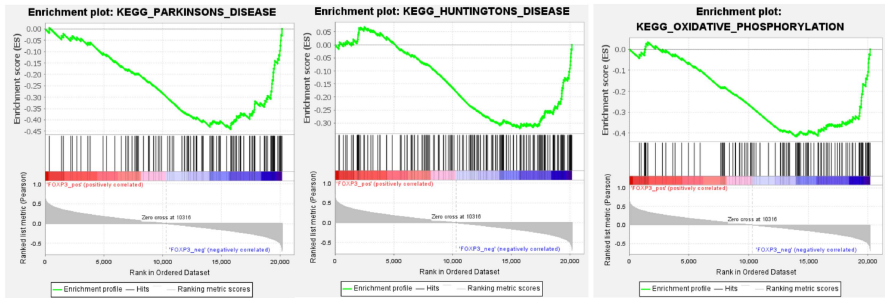

(B)

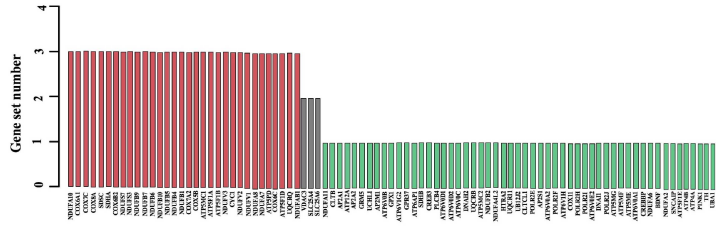

(C)

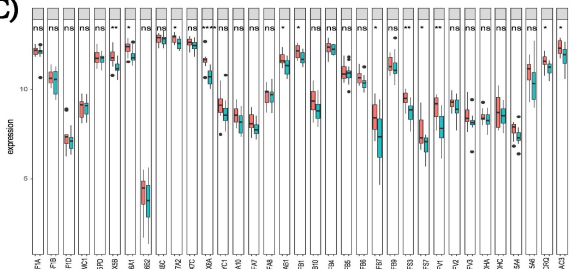

(D)

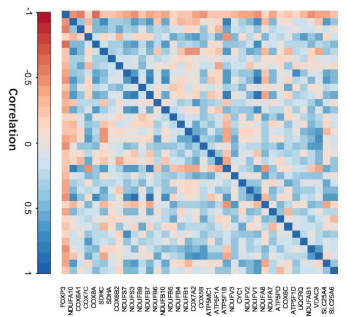

Supplement: Supplementary data [file jitc-2021-002339supp006.pdf]

(A)

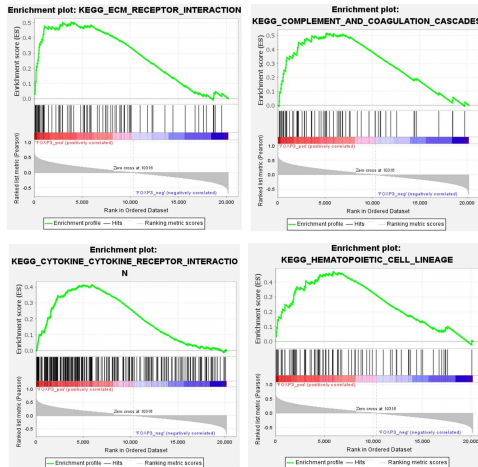

(B)

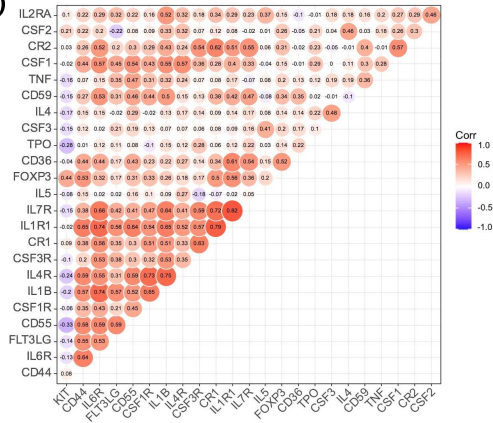

Supplement: Supplementary data [file jitc-2021-002339supp007.pdf]

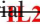

Supplement: Supplementary data [file jitc-2021-002339supp008.pdf]
